# Experimental Investigation of Impact Concrete Slab on the Bending Behavior of Composite Bridge Girders with Sinusoidal Steel Web

**DOI:** 10.3390/ma13020273

**Published:** 2020-01-08

**Authors:** Marcin Górecki, Krzysztof Śledziewski

**Affiliations:** Faculty of Civil Engineering and Architecture, Lublin University of Technology, Nadbystrzycka 40 Str., 20-618 Lublin, Poland; m.gorecki@pollub.pl

**Keywords:** composite bridge, steel–concrete composite beam, sinusoidal web, experimental study, buckling, distribution of stresses

## Abstract

Until recently, steel plate girders with corrugated steel members were used primarily as poles and girders in the construction of industrial buildings. Currently, they are also being used in the construction of bridges. Compared to traditional steel and rolled girders, steel plate girders weigh less and are more stiff, while also having a neater appearance. In this paper, the results of an experimental study are present. The aim of the study was to determine the behavior of a bridge girder with sinusoidal web geometry when subjected to a bending moment. The study was focused on a composite steel and concrete structure with pin connections, which is currently the most common solution. Three near-real scale beams were subjected to bending tests. The study found that composite corrugated-web steel beams and non-composite corrugated-web steel beams showed similar forms of failure. A reinforced concrete slab did not prevent web stress concentration at the point of connection with the flange. Furthermore, the study indicates that corrugated steel webs in bridge girders can have a much smaller thickness (less than 8 mm) compared with the traditional solution.

## 1. Introduction

Composite structures are unique in that they consist of diverse materials, and that these materials are located in specific section zones to make the best use of their stress-strain properties. This makes them some of the most interesting and effective load-bearing structural systems [1,2]. In composite steel–concrete decks, individual cross-sectional components are made of materials with different Young’s moduli [3]. These components interact through special connections [4,5,6,7].

Researchers are looking for new structural solutions to meet increasingly strict economic and operational criteria, while also adhering to the requirements of standards (including for both the ultimate limit state and serviceability limit states) [8]. These efforts have led to the emergence of a solution using corrugated-steel webs for composite, primarily steel, bridges [9,10,11,12]. Until recently, corrugated-web steel beams were used mainly as structural components of buildings, in particular as poles and girders in industrial buildings [13]. They compete with traditional steel girders and rolled profiles, mainly because of their lower weight and higher stiffness [14]. In addition, they give the structure a neater appearance [11].

In engineering practice, the geometry of I-beams with corrugated webs is designed to provide high longitudinal stiffness in the flanges with low longitudinal web stiffness due to the web shape. The bending resistance is only provided by flanges without the web. Hence, the ultimate resistance to bending depends on the geometric and material parameters of flanges alone [15,16,17,18].

Trapezoidal corrugated plates are currently the most popular solution [19,20,21,22,23,24,25,26,27,28]. Other shapes in use include sinusoidal, triangular, trough-shaped, and cellular. The most extensively explored behavior is that of steel beams with trapezoidal web geometry.

However, recent research has also investigated the static loads and load bearing capacities of girders with corrugated-plate webs [29,30,31,32,33,34]. One of the findings was that the transverse deflections of corrugated webs caused non-linearity, and increased the global displacements of girders. Furthermore, yield lines were found to form due to web shear buckling [35,36,37]. However, the above results apply to steel girders with sinusoidal webs made of 2.0, 2.5, and 3.0 mm thick plates used in the cubature construction [38]. The slenderness of such web cross-section is usually in the range 100 ÷ 600 [39]. The bridge industry places greater requirements on the size of loads to be transferred. The thickness of webs in traditional flat sheet metal girders, in bridge constructions, from constructional conditions exceeds 8 mm [40]. The advantage of reducing girder weight due to the use of corrugated steel webs, compared to the traditional girders, has provided the basis for testing girders with corrugated steel webs slightly less than 8 mm thick.

In this experimental study, authors focused on testing the bending behavior of a composite bridge girder with a sinusoidal web thickness of 7 mm. Composite steel-concrete beams were subjected to the four-point bending flexural test to determine the type of failure, load-bearing capacity, deflections, and strain redistribution, under increasing loads. The composite beams that were tested comprised of steel beams attached to reinforced concrete slabs. In addition, the results with previous tests of corrugated-web steel beams are compared. These beams had the same parameters except for the absence of the reinforced concrete slab [41].

## 2. Experimental Investigations

### 2.1. Properties of the Test Beam

This experimental study focused on three single-span steel I-beams with sinusoidal corrugated webs, stiffened with a reinforced concrete slab attached to the upper flange (marked with the symbols 1ZB, 2ZB, and 3ZB). All the tested components had the same geometric and material properties.

**Figure 1:**
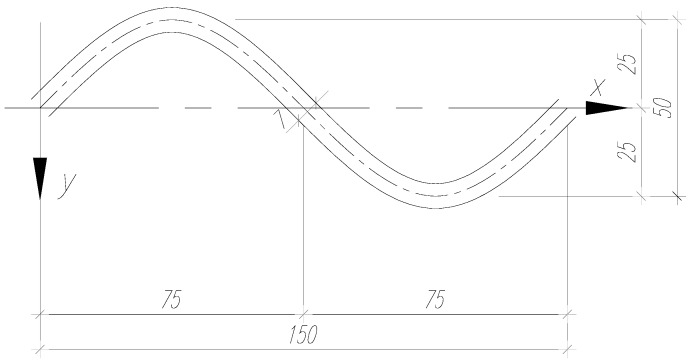
Sinusoidal corrugation geometry (mm).

It was assumed that the beams corresponded to the behavior of bridge girders [39]. Steel sections in bridge structures are larger than in industrial buildings, due to the higher load requirements. Accordingly, it was assumed that the web was 7 mm thick and 350 mm high. Figure 1 shows the sinusoidal corrugation geometry.

**Figure 2:**
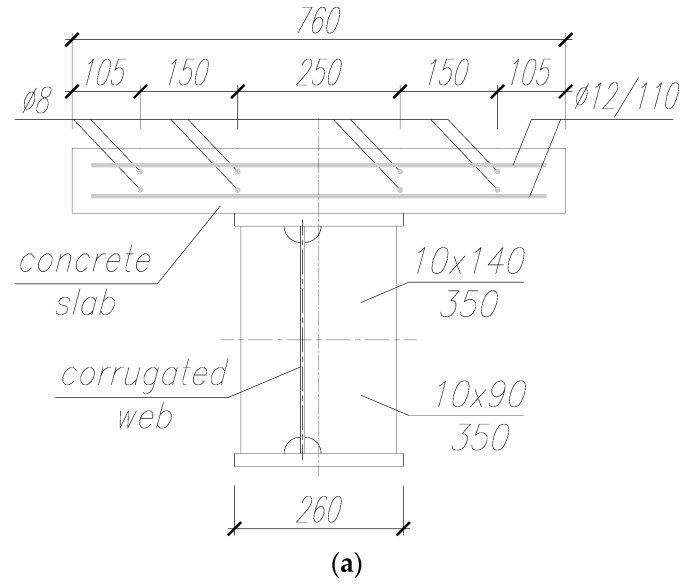

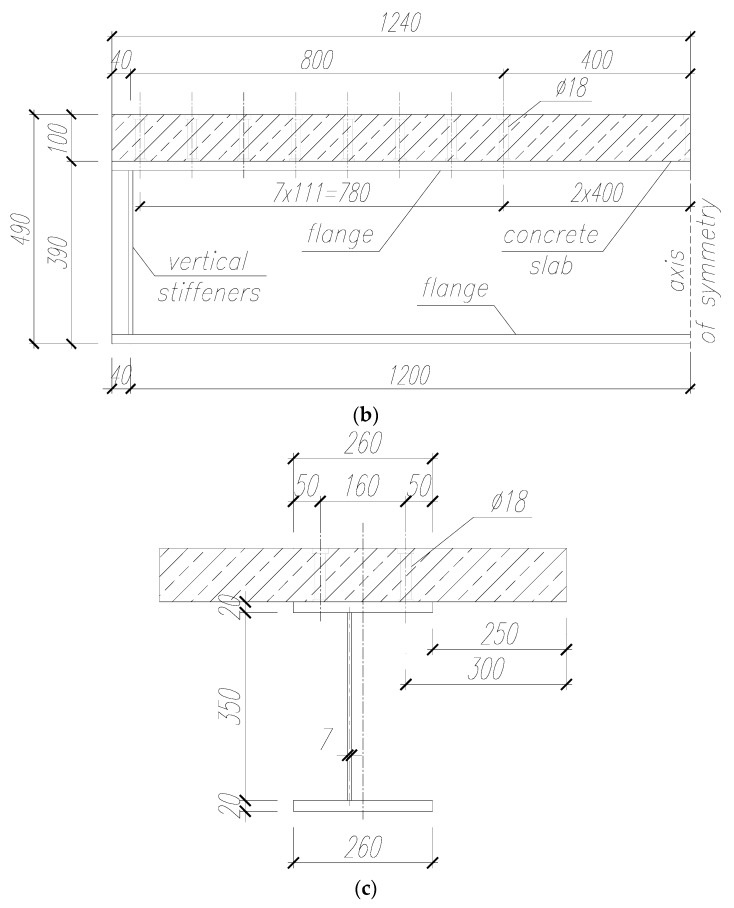
Geometric parameters of the tested beams (mm): (**a**) Transverse cross-section with the slab reinforcement arrangement; (**b**) longitudinal section with a pin arrangement; (**c**) transverse cross-section with a pin arrangement.

Figure 2 presents other geometric parameters of the tested beams. The upper and lower flange were made of flat sheets with a thickness of 20 mm, a width of 260 mm, and a total length of 2480 mm. At a distance of 40 mm from the beam ends, on each side of the web, were attached vertical rib stiffeners made of 10 mm thick flat sheets of full web height. Due to the location of corrugations relative to the flange axis of symmetry, the ribs were 90 mm wide on one side and 140 mm on the other (Figure 2a). The latter width was on both ends of the same side of the beam. The ribs marked the beam support centerlines on the test stand. There were no other web stiffeners attached along the span length.

The flanges were connected with the web using two-sided fillet welds with a thickness of 4 mm. It was also made of 4 mm thick two-sided fillet welds for rib attachment to the web and flanges.

The beam geometry adopted in this way means a slenderness of h/l ≈ 1/7 and the dimensions of the cross-section guarantee the absence of the effect of local loss of belt stability based on the following condition:

(1) $$b_{f}\leq t_{f}\cdot \varepsilon \cdot 13.9\cdot 2+22\mathrm{mm}$$

where *b_f_* is the flange width (mm); *t_f_* is the flange thickness (mm), ε is the factor depending on *f_y_*, ε = (235/*f_y_*)^1/2^; *f_y_* is the yield strength (MPa).

The shear load capacity of the beam is given by:

(2) $$V_{Rd}=\chi_{c}\frac{f_{yw}}{\gamma_{M1}\sqrt{3}}h_{w}t_{w},$$

where *χ_c_* is the reduction factor for the relevant buckling curve; *f_yw_* is the flange yield strength (MPa); *γ_M1_* is partial factor for resistance of members to instability assessed by member checks; *h_w_* is the web height; *t_w_* is the web thickness.

Based on the guidelines for the design of steel–concrete composite beams [42], the shear capacity depends only on the web parameters as in Formula (2).

The upper flange was additionally fitted with steel pins to provide connection with the reinforced concrete slab with a thickness of 10 cm and a width of 76 cm. Furthermore, were attached 34 pins (ϕ18 mm) with a center-to-center spacing of 160 mm in the transverse section and 111 mm along the beam centerline in the shear force span (Figure 2c). In the beam mid-section, between loading points, the number of connections were reduced because of the uniform bending moment expected in this location (Figure 2b). After welding the pins, the reinforcement of the slab and concrete works were performed.

The slab was provided with transverse and primary reinforcement (top and bottom bars). Transverse bars at both top and bottom located closer to the outer face of the slab had diameters of ϕ12 mm. Primary top and bottom bars were ϕ8 mm in diameter.

Concreting of the slab was done in an inverted system. The slab formwork was made and then the reinforcement bars were placed. After the spacers had been placed, the steel beam was placed with pins down in the previously prepared form. The concrete mixture was laid with surplus. Vibration ensured that the concrete was in full contact with the strip of the steel beam. The excess of concrete was removed just after the mixture was vibrated.

In addition, non-composite steel beams with the same geometric parameters as the composite plate girders with sinusoidal corrugated webs (beam 1SB) were tested.

### 2.2. Materials

All the components of the steel beam—webs, flanges, and rib stiffeners—were made of the same structural steel (S355+N). Testing also included a reinforced concrete slab made of C30/37 concrete and ribbed steel bars made of 34GS steel.

Due to possible changes in stress-strain parameters as a result of the cold rolling of the corrugated web plates, five samples of corrugated-web steel were tested. With regard to the stress-strain parameters of the bars, quality certificates were relied on as provided by the manufacturer. Table 1 shows the detailed materials data.

### 2.3. Test Setup and Instrumentation

The beams were tested in The Wroclaw Section of a Bridge, Concrete, and Aggregate Testing Center owned by the Polish Road and Bridge Research Institute.

**Figure 3:**
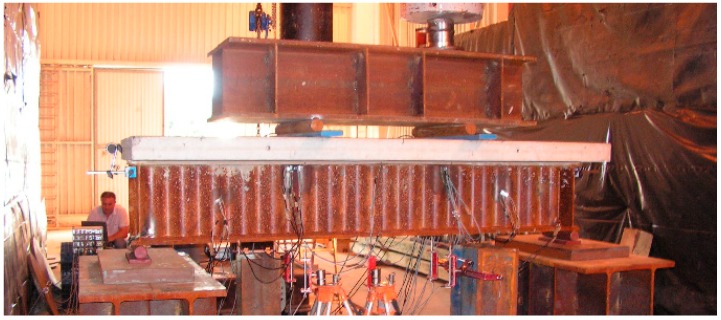
View of the typical four-point bending test setup.

**Figure 4:**
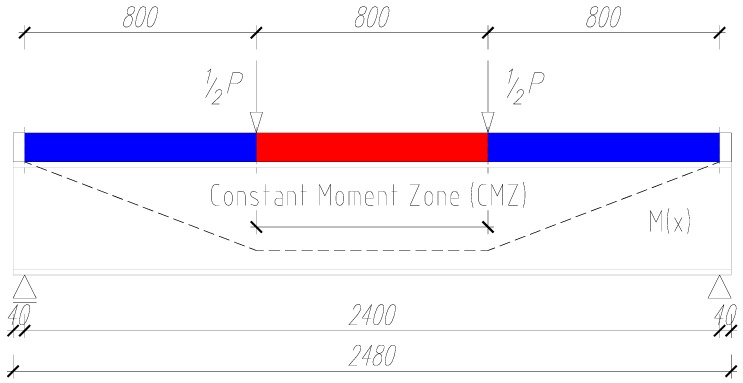
Load diagram for the tested beams—the distribution of moments along the length of the beam (mm).

The beams were subjected to a four-point bending test to obtain a constant bending moment at beam mid-span (Figure 3). One of the supports was made as a non-sliding articulated support, while the other was made as a sliding articulated support. Loads were applied at a distance of 840 mm from the test specimens’ ends. The distance between the test specimens’ ends was 800 mm (Figure 4). The external load was carried out using two actuators.

The loads were applied to the beams gradually, as concentrated forces in eight full load-unload cycles. The first cycle ranged from 0 to 200 kN, an eighth of the expected failure load. The maximum force grew by 200 kN with each subsequent cycle. After reaching the maximum load in a cycle, the specimens were unloaded to zero. After each individual cycle reached a maximum or minimum value, it was made about 180 s pauses before changing loads. During this time, readings were taken from the measuring equipment, and alterations in test specimens were observed. The cycles were repeated until the beams failed.

**Figure 5:**
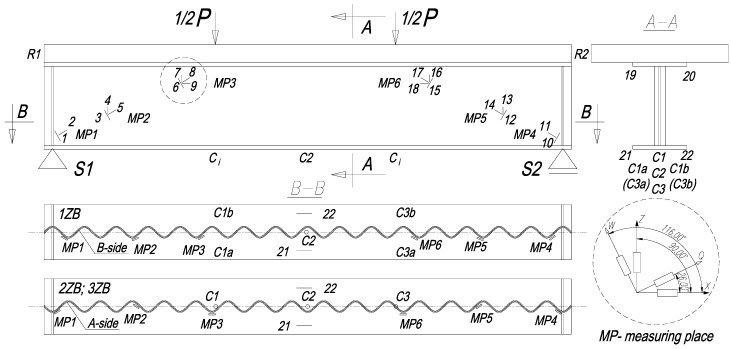
Location of the gauges in the test specimens.

The angle between the “force-support” line and girder flanges was 23.63°. Each of these lines featured three points (marked as MP1-MP6 in Figure 5) of measurement. The measurements were taken using electrical resistance strain gauges with a gauge length of 10 mm (marked as 1–22 in Figure 5). The gauges were fixed along and perpendicular to the “force–support” line. To illustrate the sides on which the gauges were fixed, sides “A” and “B” in the beams were specified. Side “A” over the support comprised the corrugation “ridge”, side “B” under the support featured the corrugation “bottom”.

On beam 1ZB the gauge was fixed to side “B”, and on beams 2ZB and 3ZB to side “A”. The gauges marked 19–22 were fixed to the lower surfaces of the upper and lower flanges. Figure 5 shows the detailed location of the sensors.

To measure the vertical displacements, inductive sensors were used (marked as C in Figure 5). In addition, dial gauges were used to measure the longitudinal displacement of the reinforced concrete slab relative to the plate girder (marked as R in Figure 5). Using magnetic indicator stands, the measurement equipment was attached to the support ribs at both ends of the specimens.

## 3. Experimental Results and Discussion

### 3.1. Load Bearing Capacity and Type of Failure

The ultimate loads at which composite beams with corrugated webs failed were 1611 kN (1ZB), 1640 kN (2ZB), and 1650 kN (3ZB). This is approximately 17% higher than the load bearing capacity of the reference beam (1SB, corrugated web, non-composite), which was 1359 kN.

**Figure 6:**
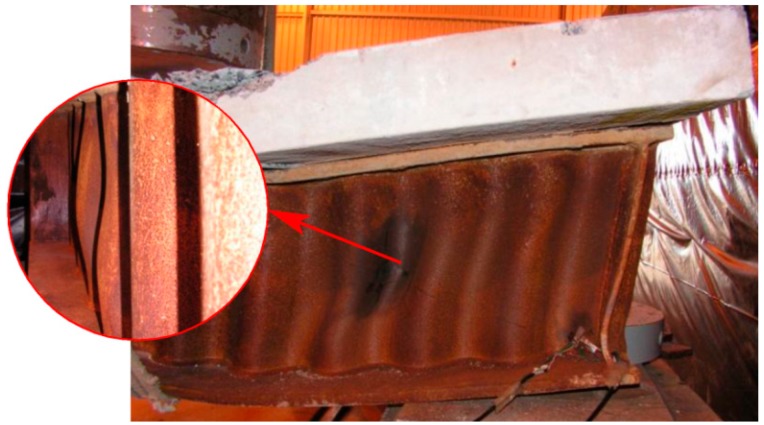
An example of a typical failed beam.

Across all loads in composite beams, there were no deformations in the steel part of the plate girder until the first concrete cracks started to appear in the compression zone. The result of damage to the reinforced concrete slab, at the point of transferring the external load, was the destruction of the flanges and ultimately the web. The appearance of the failed web clearly demonstrates global web buckling. Corrugation deformations fade towards the end of the beam. Figure 6 shows an example of a typical failed beam.

The failure occurred abruptly in the zones under constant transverse loads, in which the bending moment decreased towards the support. The largest deformations are visible along the section close to the point of loading. This means that the beams had reached their ultimate strength due to the large bending moment and shear force occurring at the same time.

It was considered that the formation of yield lines in shell structures due to corrugated-web buckling causes a sudden load on the flanges. The result is a plastic hinge in flanges and beam failure. The reason for the plastic hinge is that flanges have a relatively low in-plane resistance to bending. In addition, the reinforced concrete slab does not provide an adequate distribution of stress, which concentrates in the web at its point of connection with the flange.

**Figure 7:**
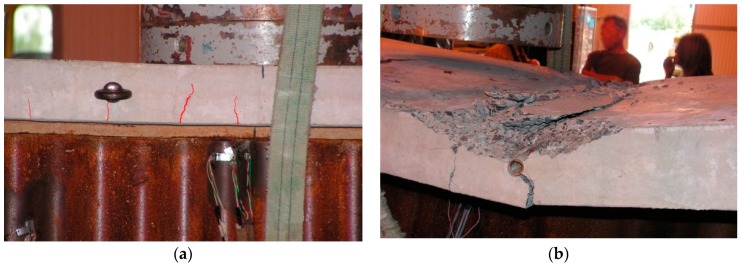
Reinforced concrete slab damage: (**a**) Crack morphology; (**b**) concrete crushing.

Ultimately, the beam failed because of concrete crushing within the loading zone (Figure 7), and the absence of the shear-force balancing effect in the web.

**Figure 8:**
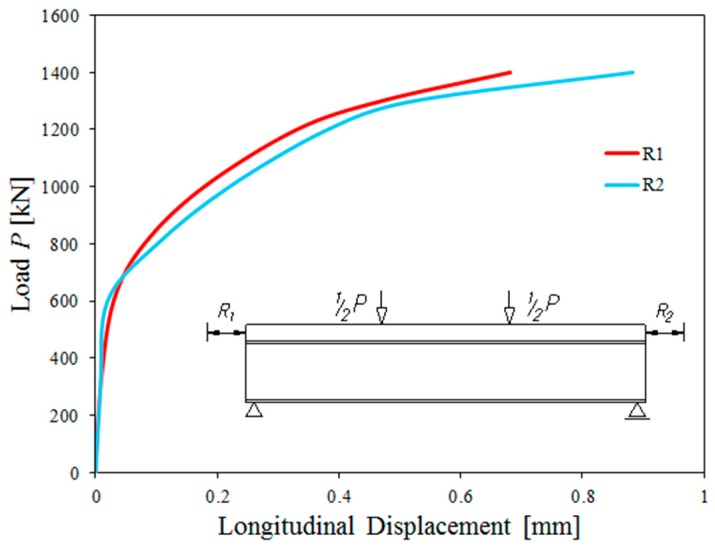
Typical reciprocal longitudinal displacement of the reinforced concrete slab and beam at all load ranges.

One of the reasons why the composite beams failed might also have been a detachment between the reinforced concrete slab and the steel beam due to the connection shear failure [40]. Therefore, the reciprocal longitudinal displacements of the slab and beam on each side of the component were measured (Figure 8).

At a load of 1400 kN (85% of the failure load), reciprocal displacements did not exceed 0.9 mm. At this load, the displacement sensors were removed for safety reasons. Such small reciprocal displacements, even after cracking, representing 0.07% of vertical displacements, proved that the attachment was complete [43]. After the completion of the destructive tests, visual tests were carried out on the basis of which it was found that the pins were not cut.

### 3.2. Load–Displacement Relationship

Deflections were measured by assessing the vertical displacement of the lower surface of the lower flange. For beam 1ZB, the lower flange displacements were recorded at five measurement points. In the midspan, one sensor was fixed at the vertical cross-section centerline. In the cross-sections exposed to external loads, two sensors were installed on the outer edges of the lower flange. It was assumed that the arithmetic mean of the displacements of measurement points in the same transverse cross-section of the beam would be a reliable measure for further analysis.

The measurements of the vertical displacements of beams 2ZB and 3ZB took place at three measurement points within cross-sections subjected to concentrated forces, and at beam midspans. All the sensors were located in the plane containing the transverse cross-section centerline of the beam. Table 2, Table 3 and Table 4 presents the results. For technical reasons, for the 2ZB and 3ZB beams, no displacement measurements were taken for the last cycle just before destruction.

All the tested beams with corrugated webs and reinforced concrete slabs exhibited constant stiffness until the concrete started to crack. In the next phase of testing, uniformity between the displacements of beams 2ZB and 3ZB were observed. Beam 1ZB exhibited deflections which were about 40%–50% smaller as early as in the first and second cycles of loading. In subsequent cycles this difference decreased to 30%–40%.

**Figure 9:**
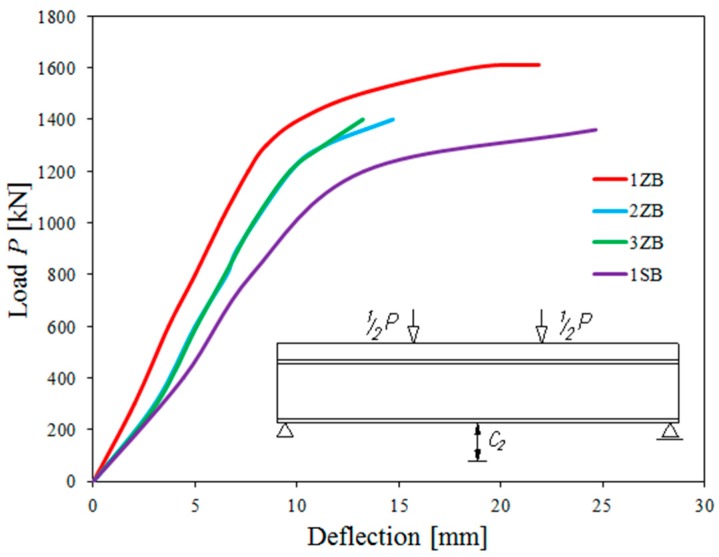
Vertical displacements of tested components at midspan (sensor C2 measurement).

Figure 9 shows the relationship between the composite-beam midspan deflection and the applied external load. For comparison, it includes corresponding charts for the reference beam (1SB).

The study proved that composite beams with corrugated webs provided higher cross-sectional stiffness and strength than non-composite beams with corrugated webs. During the tests, the transverse stiffness of the composite beams substantially reduced the global displacement of the system. Vertical beam displacements in the elastic deformation region were low, at about 20% relative to the deflections at the limit load.

**Figure 10:**
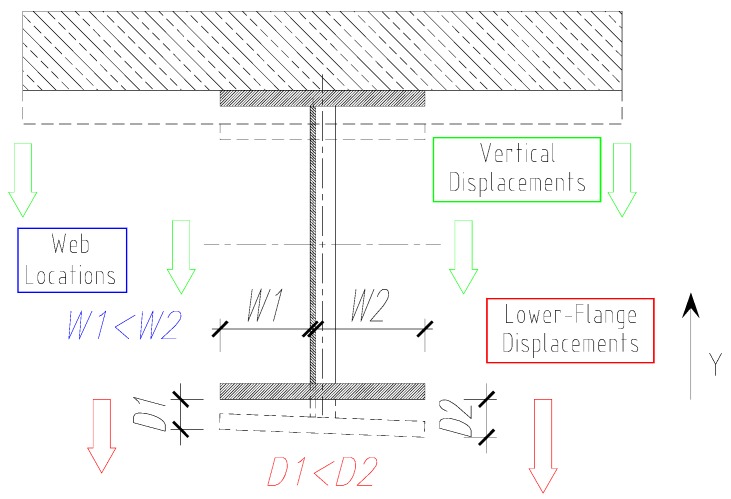
Lower-flange displacements in the transverse plane at the point of external load.

It was also noted that the cross-sectional displacement of the lower flange alone depends on the web location in the relevant cross-section. Vertical displacements of the lower flange varied along its width. They were bigger on the side of the web on which the web was located farther away from the flange edge. Despite the flange’s exposure to tension forces, lower-flange displacements in the transverse plane at the point of concentrated forces grew in a non-uniform fashion. In the initial load cycles, the displacements at the end edges of the lower flange, within the same transverse cross-section, differed by about 30% (Figure 10).

This effect was due to the flange projection changing along with the web shape. As the load increased, with the decreasing web impact on flange stiffness, this difference grew systematically smaller until the test ended. At a load of 1600 kN, this difference was 2% for measurement point C1 and 10% for measurement point C2.

### 3.3. Results of Strain (Stress) Measurements

Strain values grew the fastest and were the highest along and perpendicular to the “force–support” line. The most representative strain readouts from individual measurement points are provided in Table 5 and Table 6.

The results of the strain tests on the beams with sinusoidal webs indicate that such measurements are highly sensitive to a number of factors. These include small shifts in the measurement points, their position in respect of the corrugation ridges, and their location on the “front” or “back” side of the web. This makes it difficult to compare our results with corresponding studies involving other test specimens. The same effect applies to strains recorded for the same test specimen but with gauges located symmetrically to the beam’s axis of symmetry. A strain value measured by the gauge on one end of the beam might differ (in value and in sign) from a strain value in the corresponding point on the other end of the beam.

For instance, gauges 4 and 5 indicated strain values reflecting compressive stress, while gauge 3 showed values representative of tensile stress. In addition, were see that signs change depending on how large the external load is, e.g., at measurement points 4 and 13, and 6 and 15. This proves that their character depends on the surface of the web. In addition, it can be observed that the fastest increase in deformation values, as well as the highest deformation values are on the “force-support” line and perpendicular to it. This phenomenon is also reproduced in the form of the final destruction of the test elements, in the course of the plastic refraction line (Figure 6).

**Figure 11:**
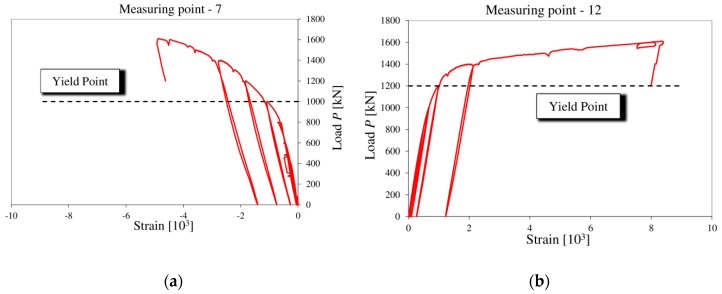
Strain distribution after the yield point was exceeded: (**a**) Measurement point 7; (**b**) measurement point 12.

Strain gauge readouts indicated that the yield point in the web was exceeded (measurement point 7) under the point of loading at about 1000 kN (Figure 11a). A further increase in load did not, however, cause the component to fail due to the tensile strength of steel’s being exceeded in this zone.

**Figure 12:**
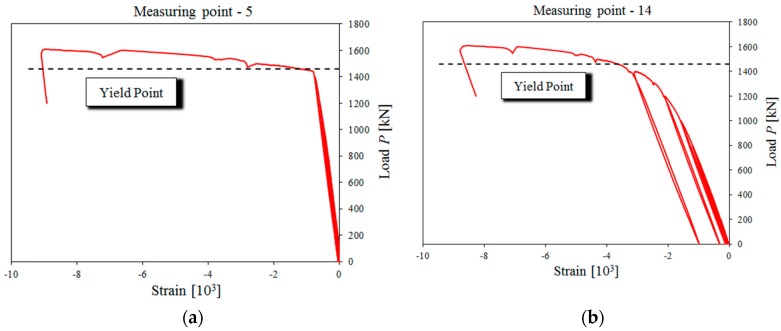
Strain increase at: (**a**) Measurement point 5; (**b**) measurement point 14.

Another location which exceeded the yield point was the shear zone on the other end of the beam. The tensile stress was identified there (measurement point 12). After the yield point, at about 1200 kN (Figure 11b), a sudden increase in strain were observed. At a load of 1460 kN, the formation of a yield line caused compressive stresses to increase at measurement points 5 and 14 (Figure 12). This confirms the nature of the destruction of the support zones.

During the experiment, the upper-flange stresses did not reach the yield point, and in the lower flange the yield point occurred at about 1500 kN.

## 4. Concluding Remarks

In this study, authors analyzed the behavior of a composite steel–concrete I-beam bridge girder with sinusoidal web geometry when subjected to a four-point loading test. It was that composite girders with corrugated webs could transfer large loads without web stiffeners. The absence of ribs in corrugated-web beams, especially under the points of loading, did not cause local web buckling. The only effects were the increased strain values, as recorded by the strain gauges fixed near the load areas.

The results of the studies make it possible to formulate the following conclusions:

- Steel–concrete composite beams with corrugated-web and non-composite corrugated-web steel beams showed similar forms of failure. For steel beams with corrugated webs attached to reinforced concrete slabs, the ultimate strength in the four-point bending test determined the ultimate strength of the entire system.
- For the elements tested, the increase in deflections in the elastic range is small, which is due to the stiffness of the whole system, while a rapid increase in deflections is noticeable during the beam operation in the range of appearance of plastic deformations in the corrugated web.
- The distribution of deformations changes significantly on a small area of the corrugated web plate on the scale of the whole element. Tensile strain occurred on one side of the web, while compressive strain was visible on the other side. Exceeding the yield strength did not cause immediate failure of the beam.
- The reinforced concrete slab was not able to increase the load area enough to avoid the stress concentration in the web at contact with the flange, causing high deformation values. As a consequence, the bending moment is transmitted mainly through the flanges and the shear stress through the web.

## Figures and Tables

**Table 1:** Stress-strain parameters of steel.

Steel Grade	Yield Point [MPa]	Average Tensile Strength [MPa]	Elongation to Failure [%]	Young’s Modulus [MPa]	
				Longitudinal	Transverse
Profile Steel	434	512	27.2	2.02 × 10^5^	0.78 × 10^5^
Reinforcement Steel	410	550	40.1	2.10 × 10^5^	-

**Table 2:** Deflection of 1ZB beam (Unit: Mm).

Measure Points	Load-Unload Cycle [kN]								
	0	300	0	800	0	1400	0	1600	0
C1a	0.00	1.41	0.19	3.74	1.36	8.43	2.63	15.58	11.94
C1	0.00	1.68	0.29	4.16	1.60	9.01	2.90	15.74	11.76
C1b	0.00	1.94	0.40	4.58	1.83	9.59	3.16	15.89	11.58
C2	0.00	2.02	0.30	5.01	1.71	10.11	3.28	18.68	13.90
C3a	0.00	1.62	0.21	4.08	1.35	9.16	2.94	18.18	14.66
C3	0.00	1.93	0.33	4.58	1.67	9.87	3.32	19.10	15.18
C3b	0.00	2.25	0.46	5.08	1.99	10.57	3.69	20.02	15.96

**Table 3:** Deflection of 2ZB beam (Unit: Mm).

Measure Points	Load-Unload Cycle [kN]						
	0	300	0	800	0	1400	0
C1	0.00	2.65	--	5.85	2.17	13.63	6.74
C2	0.00	3.02	--	6.55	2.49	14.70	7.06
C3	0.00	2.98	--	6.18	2.56	14.11	7.09

**Table 4:** Deflection of 3ZB beam (Unit: Mm).

Measure Points	Load-Unload Cycle [kN]						
	0	300	0	800	0	1400	0
C1	0.00	3.14	0.73	6.03	2.91	12.46	7.92
C2	0.00	3.10	0.64	8.31	2.42	13.22	7.40
C3	0.00	3.10	0.76	6.20	2.44	12.43	7.43

**Table 5:** Typical strain on the measurement points perpendicular to the “force–support S1” line for beam 1ZB (Unit: ‰).

Load [kN]	Measurement Points								
	X	Z		Q			W		
	9	4	7	2	5	8	1	3	6
1400	--	−0.07	−2.71	−1.11	−0.75	−0.56	0.54	1.20	0.36
1611.27	--	−0.24	−4.87	−2.06	−8.96	−0.83	1.10	1.76	0.62

Note: Letters X, Z, Q, and W refer to strain-gauge directions at the measurement points according to Figure 5.

**Table 6:** Typical strain on the measurement points perpendicular to the “force–support S2” line for beam 1ZB (Unit: ‰).

Load [kN]	Measurement Points								
	X	Z		Q			W		
	18	13	16	11	14	17	10	12	15
1400	0.23	−0.14	−1.36	−1.69	−1.51	−0.86	0.90	1.99	−0.27
1611.27	0.64	0.33	−2.59	−7.18	−4.24	−1.75	2.85	8.34	0.03

Note: Letters X, Z, Q, and W refer to strain–gauge directions at the measurement points according to Figure 5.
